# How to Sell Waste Paper

**Published:** 1916-02-26

**Authors:** 


*To the Editor of* The Hospital.

### How to Sell Waste Paper.

*To the Editor of* The Hospital.

Sir,—I should be greatly obliged for information how to sell waste paper. Would it pay to have it roughly sorted in the various offices, selling newspapers, writing-paper, etc., separately? The threat to do so has made our present buyer double his price for it unsorted. Up to the present he has posed as a philanthropist, collecting our waste paper almost as an act of charity; inquiry, therefore, has made him doubly philanthropic. I should be grateful for any information which will enable me to continue to improve this good man.—Yours, etc.,

A Provincial House Governor and Secretary.

February 15, 1916.

